# Cognitive Bias: Phylogenesis or Ontogenesis?

**DOI:** 10.3389/fpsyg.2022.892829

**Published:** 2022-07-27

**Authors:** Hans Van Eyghen

**Affiliations:** ^1^Faculty of Religion and Theology, VU Amsterdam, Amsterdam, Netherlands; ^2^Tilburg School of Catholic Theology, Tilburg University, Tilburg, Netherlands

**Keywords:** cognitive bias, evolutionary psychological science, agency detection, theory choice, bias

## Introduction

The literature on cognitive biases is vastly expanding. The contribution of cognitive biases to the formation of beliefs and the process of believing (cf. Seitz et al., 2016; Angel, 2017;) is well documented. Well-documented examples are the confirmation bias (Mahoney, 1977), and the self-serving bias (Campbell and Sedikides, 1999). Most of the literature focuses on testing the existence and salience of various cognitive biases. Fewer authors focus on the *causes* of cognitive biases. This paper compares two mutually conflicting accounts of how cognitive biases arise. A first argues that (most) cognitive biases are part of the general human cognitive makeup, which is innate or emerges as humans mature. A second argues that cognitive biases are acquired throughout a human's lifespan and development. Below, I present examples of both accounts and reasons favoring each of both accounts.

A large number of definitions of “cognitive bias”^1^have been proposed. Some regard cognitive biases as *epistemic;* for example, as a “systematic pattern of deviation from norm or rationality in judgment” (Hasselton et al., 2005) or a “top-down, subjective directed perception” (Kahneman and Tversky, 1972). Others regard cognitive biases as forms of automatic cognition; for example as “automatic information processing.” (Shiffrin and Schneider, 1977) or “Information processing without attention” (Payne and Gawronski, 2010). The definition used throughout this paper considers cognitive biases as skewed perceptions or skewed belief-formation. Because of cognitive biases, humans have a tendency for cognition to go in a particular direction, thereby giving rise to recurring patterns.

On most accounts of bias, humans usually remain unaware of their operations. While humans can be made aware through introspection or external information, most humans have a hard time explaining why their cognition is skewed in a particular direction.

In the next two sections I summarize two prominent accounts of how humans end up with cognitive biases. Caution should be made that the lines between both accounts are not clear-cut. Defenders of a phylogenistic account of cognitive bias usually agree that at least some biases are acquired throughout one's lifespan. The same holds for defenders of an ontogenistic account, although they allow for rather few innate cognitive biases. The difference is thus primarily one of focus, arguing that *most* or the *most salient* cognitive biases should be explained phylogenistically or ontogenistically.

## Bias in Philogenesis

A first account of the causes of cognitive biases argues that most cognitive biases result from the way the human mind is structured species-wide. Because of the way human minds and brains are, a number of biases arise. Some of these are innate, other gradually manifest as human brains mature^2^. On this account, cognitive biases are ultimately encoded in the genome, which ultimately traces back to various evolutionary pressures.

While (some) biases require being triggered by sensory input on phylogenetic accounts, the response to that input is “preprogrammed” or predetermined by the human cognitive make-up. Sensory input is thus not the main determinant of why the bias originated.

One example of a bias that is commonly explained in this way is the bias to find food, heat and shelter (see Friston et al., 2012). Because most organisms require these very shortly after birth, the bias needs to be hard-wired or innate.

This line of reasoning has been applied to explain a cognitive bias for the detection of agents. Various authors have noted that humans are prone to see agency in natural or material phenomena. A famous example is the Heider-Simmel simulation (Heider and Simmel, 1944). The tendency to see agency even when such agency is absent under closer inspection would lie at the roots of animism or even religious beliefs according to some (e.g., Barrett, 2004).

Stewart Guthrie argues that our proneness to promiscuously perceive agency has its roots in evolutionary pressures. He argues that it was evolutionary beneficial for our distant ancestors to be highly sensitive for agency. Hypersensitivity compares favorably to lower sensitivity because low sensitivity increases the risk of missing one predator or rival human. Given the high threat, odds of doing so had better be diminished. Hypersensitivity diminishes the risk of missing predators or rivals greatly and only has minor costs (i.e., loss of time and energy) by comparison. For this reason, natural selection selected for a proneness to see agency on very limited evidence (Guthrie, 1993).

Guthrie does not discuss how evolutionary pressures led to a change in the human genome, which in turn leads to a cognitive bias. It is clear, however, that on Guthrie's account a bias for agency detection is not acquired throughout one's lifespan but part and parcel of the kind of mind or brain humans are endowed with. His account is therefore a clear example of a phylogenistic account of a cognitive bias.

## Bias in Ontogenesis

Phylogenistic accounts of cognitive biases are arguably dominant in evolutionary psychology. Authors in cognitive neuroscience in particular tend to favor a different account where (most) cognitive biases are not hard-wired in the human brain but acquired because of the way the human mind engages with its environment. On such an ontogenetic account, humans are not born with the bias, not even in potentia.

Uncontroversial acquired biases are cultural biases like different responses to smiles or different levels of trust in various cultures (cf. Guiso et al., 2009). Skinner et al. (2020) argue that in-group biases are likely acquired by exposure to positive or negative responses to novel adults from out-groups. These biases are not ingrained within innate cognitive structures or the result of normal development thereof. Instead, they crucially depend on exposure to specific (sensory) input.

Ontogenetic accounts of the agency bias have been defended. Marc Andersen argues that the bias does not result from our evolved nature but depends on preexisting beliefs or priors that makes the presence of invisible agents more likely. Especially religious beliefs that invisible spirits or gods exist would raise the expectation of experiencing such beings in subjects. As a result, subjects with those beliefs would display a higher proneness to (over) detect agency (Andersen, 2017)^3^.

Acquired biases need not be culturally specific. Some biases that recur cross-culturally can be the result of interactions with a similar environment by subjects with similar cognitive functions. Elsewhere I argued that a bias for agency (over) detection could result from common human processing of agents (Szocik and Van Eyghen, 2021). Given that human brains have limited computing power, brains have a hard time of keeping track of all features that indicate agency. Therefore, it is more efficient to focus on one or a few clear indicators of agency, like self-propelled movement and/or complex patterns. While such a simplification^4^ allows human brains to quickly and efficiently detect agents, the flipside is increased proneness for misidentification. As Guthrie and other note, inanimate things occasionally appear to engage in self-propelled motion, like leaves rustling in the wind or branches falling from trees. A brain that focuses on self-propelled motion as an indicator of agency will therefore be biased to connect such movement to agency. Multiple cultural environments could give rise to the same simplification and therefore the bias could arise cross-culturally.

## Ontogeny or Phylogeny?

Few, if any, authors are exclusivists with regard to a phylogenetic or ontogenetic genesis of biases. As noted, evolutionary psychologists lean toward accepting that more biases have a phylogenetic origin. Cognitive neuroscientists tend to accept a very limited number of biases of phylogenetic origin. As a result, there are conflicting accounts of a number of biases like the agency bias.

The existence of conflicting accounts suggests that both are underdetermined by the empirical data. On both accounts, biases have a similar phenomenology with recurring patterns in cognition that are hard to override. Nonetheless, both accounts predict some different empirical observations concerning biases. On an ontogenetic account, we would expect more variation depending on the (cultural) environment. An ontogenetic account also predicts more individual variation within groups.

Another observable difference is that phylogenetic biases are harder to override. Phylogenetic biases are regarded as a default state of the human cognitive system. While subjects can override this default state (for example, through rational deliberation or cognitive aides), the default state never disappears. When overriding factors lose their force, the default state will resurface. For example, some have argued that a bias to think of things teleologically or for a purpose resurfaces when subjects are put under time pressure (Kelemen et al., 2013) or forget about overriding information (Lombrozo et al., 2007). Phylogenetic biases thus repeatedly intrude or re-intrude on cognition. Given that ontogenetic biases are more malleable and display more variation, we would expect less intrusions of this kind if the bias were acquired^5^.

Other evidence favoring a phylogenetic account would be evidence that a bias is present in very young children. Young children had little or almost no exposure to the sensory data needed for biases to take hold on an ontogenetic account. Therefore, evidence of a bias at a very young age is better explained as the result of the innate structures of their minds. A caveat must be made that sensory input already makes a substantial impact on children's minds from a very young age.

Contrary to what some suggest, evidence for a bias in non-human animals (e.g., Blanchard et al., 2014) does not necessarily favor a phylogenetic account. Like humans, some animals have learning-capacities that enable them to acquire biases through repeated exposure to similar sensory stimuli.

In the absence of evidence favoring either a phylogenetic or ontogenetic account, theoretical virtues can play a decisive role.

An ontogenetic account is usually more parsimonious because it need not postulate anything beyond the plasticity of the human mind. An ontogenetic account also has more predictive power given that acquisitions of biases are easier to track than innate cognitive structures or evolutionary pressures.

## Conclusion

I have surveyed two rivaling accounts of human cognitive bias. One puts its origins in the development of the human species and claims that the bias is part and parcel of natural human cognitive operations. The second states that the bias was acquired at some point in a human's development through specific sensory input and processing thereof. While I discussed a number of empirical traits that can help distinguish phylogenetic from ontogenetic biases, I argued that such evidence is often hard to come by. In the absence of such evidence, parsimony and predictive power generally favor an ontogenetic account.

## Author Contributions

The author confirms being the sole contributor of this work and has approved it for publication.

## Funding

This paper was funded by the Volkswagen Foundation, Siemens Healthineers, and the Betz Foundation.

The authors declare that this study received funding from Siemens Healthineers. The funder was not involved in the study design, collection, analysis, interpretation of data, the writing of this article or the decision to submit it for publication.

## Conflict of Interest

The author declares that the research was conducted in the absence of any commercial or financial relationships that could be construed as a potential conflict of interest.

## Publisher's Note

All claims expressed in this article are solely those of the authors and do not necessarily represent those of their affiliated organizations, or those of the publisher, the editors and the reviewers. Any product that may be evaluated in this article, or claim that may be made by its manufacturer, is not guaranteed or endorsed by the publisher.

## Footnotes

^1^As the term designates, I will not take various biases for action into account in this paper.

^2^Some argue that phylogenetic enlargement of the brain could be related to the evolution of human beliefs (Seitz and Angel, 2020). The argument applies to the evolution of cognitive biases on a phylogenetic account as well.

^3^Andersen's account fits in a broader cognitive framework where the human mind is regarded as a self-organizing entity that builds an internal model of the world. The internal model holds statistical information on the likelyhood of encountering certain entities. The information can be updated when humans encounter mismatches between the inner model and sensory input (Friston, 2010).

^4^The simplification resembles feature reduction in statistical modeling in machine learning.

^5^Ontogenetic biases could, however, also intrude on cognition if the biases get firmly entrenched. For a discussion, see: Szocik and Van Eyghen (2021).
